# Capsule Type and Amount Affect Shedding and Transmission of *Streptococcus pneumoniae*

**DOI:** 10.1128/mBio.00989-17

**Published:** 2017-08-22

**Authors:** M. Ammar Zafar, Shigeto Hamaguchi, Tonia Zangari, Michael Cammer, Jeffrey N. Weiser

**Affiliations:** a Department of Microbiology, New York University School of Medicine, New York, New York, USA; b Division of Advanced Research Technologies, New York University School of Medicine, New York, New York, USA; Mississippi State University

**Keywords:** *Streptococcus pneumoniae*, transmission, bacterial shedding, capsular polysaccharide, host-pathogen interactions, influenza A, pneumococcus

## Abstract

The capsular polysaccharide (CPS) of *Streptococcus pneumoniae* is characterized by its diversity, as it has over 95 known serotypes, and the variation in its thickness as it surrounds an organism. While within-host effects of CPS have been studied in detail, there is no information about its contribution to host-to-host transmission. In this study, we used an infant mouse model of intralitter transmission, together with isogenic capsule switch and *cps* promoter switch constructs, to explore the effects of CPS type and amount. The determining factor in the transmission rate in this model is the number of pneumococci shed in nasal secretions by colonized hosts. Two of seven capsule switch constructs showed reduced shedding. These constructs were unimpaired in colonization and expressed capsules similar in size to those of the wild-type strain. A *cps* promoter switch mutant expressing ~50% of wild-type amounts of CPS also displayed reduced shedding without a defect in colonization. Since shedding from the mucosal surface may require escape from mucus entrapment, a mucin-binding assay was used to compare capsule switch and *cps* promoter switch mutants. The CPS type or amount constructs that shed poorly were bound more robustly by immobilized mucin. These capsule switch and *cps* promoter switch constructs with increased mucin-binding affinity and reduced shedding also had lower rates of pup-to-pup transmission. Our results demonstrate that CPS type and amount affect transmission dynamics and may contribute to the marked differences in prevalence among pneumococcal types.

## IMPORTANCE

*Streptococcus pneumoniae*, a leading cause of morbidity and mortality, is readily transmitted, especially among young children. Its structurally and antigenically diverse capsular polysaccharide is the target of currently licensed pneumococcal vaccines. Epidemiology studies show that only a subset of the >95 distinct serotypes are prevalent in the human population, suggesting that certain capsular polysaccharide types might be more likely to be transmitted within the community. Herein, we used an infant mouse model to show that both capsule type and amount are important determinants in the spread of pneumococci from host to host. Transmission rates correlate with those capsule types that are better at escaping mucus entrapment, a key step in exiting the host upper respiratory tract. Hence, our study provides a better mechanistic understanding of why certain pneumococcal serotypes are more common in the human population.

## INTRODUCTION

*Streptococcus pneumoniae* (the pneumococcus) remains a leading cause of infectious morbidity and mortality (1). The pathogenesis of pneumococcal disease begins by colonization of the mucosa of the upper respiratory tract (URT) of its obligate human host (2, 3). Colonization is especially common in young children, who typically have serial and overlapping episodes of carriage by different pneumococcal strains. Colonization by an isolate of one serotype (or type) generally does not confer cross protection to strains of other pneumococcal types (4). The basis of this antigenic diversity is a thick layer of capsular polysaccharide (CPS), which inhibits opsonophagocytic killing and is the main virulence determinant of the pneumococcus (5). Encapsulation, through expression of genes in the *cps* locus, also prevents pneumococci from becoming entrapped in the mucus layer that continuously sweeps the URT, thus allowing the organism to access and stably colonize the underlying epithelium (6).

Pneumococcal CPS is immunogenic, and the improved antibody response to CPS when conjugated to a protein has allowed for the development of vaccines now in widespread use. These pneumococcal conjugate vaccines (PCV) have been effective at preventing most invasive disease, even though they currently contain a limited number (10 to 13) of the >95 currently known types, as only a few types (members of serogroups 6, 14, 19, and 23) are commonly carried and responsible for the majority of pneumococcal disease (7). The dominance of these types has been largely stable over time and consistent among different populations across the world (before the introduction of PCV [8]). It remains unclear why certain types predominate when immune pressure is expected to promote increased diversity. It has been suggested that the expression of common CPS types is associated with greater fitness during carriage (9). Strains of many types, however, demonstrate similar levels of URT colonization in animal models (6). In addition, the immune pressure from the use of PCV has led to a diversification of types (serotype replacement), suggesting that a very wide array of pneumococcal types is generally fit for colonizing the human host (10–12). A further issue that might impact the prevalence of pneumococcal types is intra- and interstrain variation in the quantities of CPS per cell, including among different strains of the same type (13). The thickness of the CPS layer affects interactions with the mucus layer of the URT and access of surface-exposed bacterial adhesins to host receptors on epithelial cells and, thereby, may contribute to the differential fitness for colonization among pneumococcal types (6, 14, 15).

Much of the efficacy of PCV has been attributed to their effect in reducing the acquisition of carriage in immunized individuals (16). The reduction in numbers of carriers greatly amplifies the effectiveness of vaccination by reducing transmission to unvaccinated population (“herd immunity”) (17). This protection of unvaccinated individuals points to the critical role of host-to-host transmission in the overall prevention of pneumococcal infection. In order to better define the biological basis of transmission, we, and others, have modeled pneumococcal transmission and the effect of anti-CPS immunity using infant mice (18–20). Transmission is thought to involve close contact with nasal secretions from carriers, and when modeled in mice, the burden of bacteria shed by colonized hosts determines the chance of transit to a new host (21, 22). The purpose of the current study is to examine the contribution of CPS to shedding and transmission using an infant mouse model (18). By constructing capsule switch and promoter variants in an otherwise-isogenic background, we demonstrate that differences in both the types and the amounts of CPS affect shedding and, as a result, impact the frequency of transmission. Our findings suggest that the effect of CPS type and amount on shedding and transmission may be an important factor in the varied prevalence of pneumococcal types.

## RESULTS

### Pneumococcal factors involved in differences in shedding among strains.

**FIG 1:**
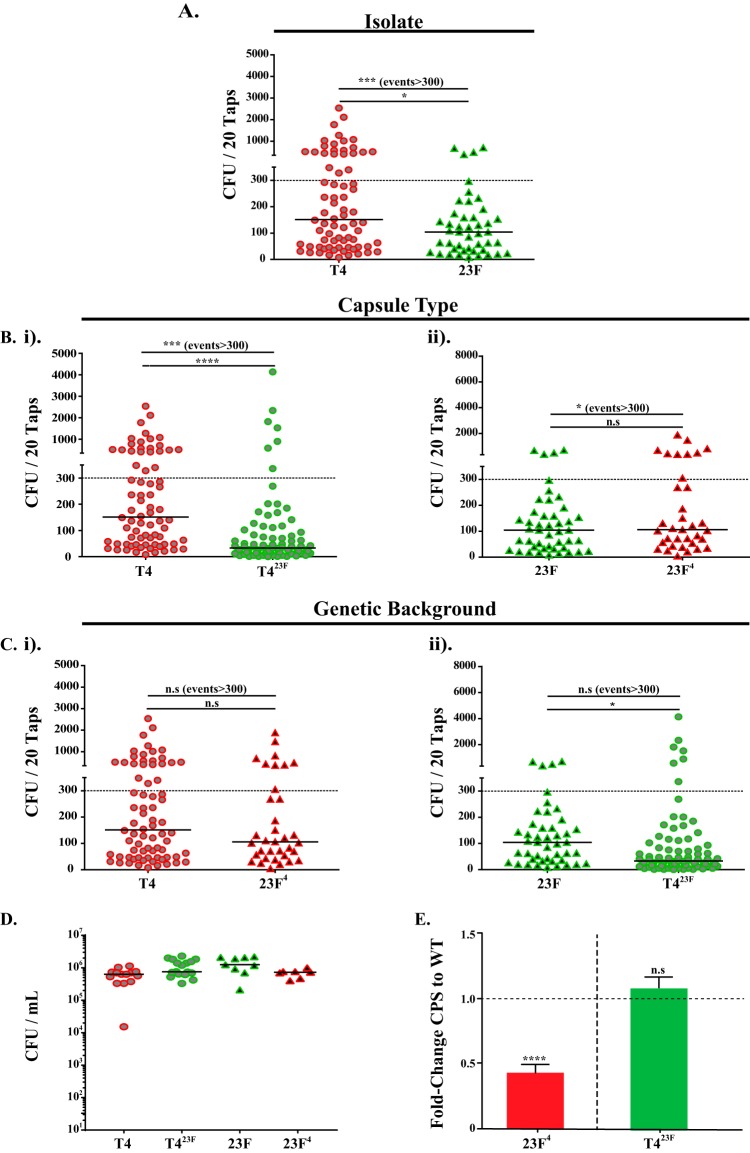
Bacterial factors in strain-to-strain differences in pneumococcal shedding. (A) Comparison of levels of shedding between two different isolates (18). Pups were challenged intranasally (i.n.) at 4 days of age with either a type 4 or a type 23F isolate. Bacteria from daily shedding were collected and quantified from nasal secretions on days 5 to 9. Median values are indicated, and each symbol represents the CFU measured from a single pup. The dashed line represents the 300-CFU threshold described in Results. (B) The role of capsular polysaccharide (CPS) type in shedding was determined by comparing the T4 (i) or 23F (ii) isolate with its capsule switch derivative expressing the other CPS type (indicated in superscript). Strains expressing type 4 and 23F CPSs are shown in red and green, respectively. (C) The role of genetic background in shedding was determined by comparing the isolate (type 4 or 23F) to the CPS switch construct in the other genetic background expressing either the T4 (23F^4^) (i) or 23F (T4^23F^) (ii) CPS type. (D) Colonization densities of the two isolates and their capsule switch derivatives obtained from lavage fluids of the upper respiratory tracts (URTs) of pups at 9 days of age. Median values are shown. (E) Relative levels of expression of CPS in capsule switch derivatives compared to the type 4 or 23F isolate using a quantitative capture ELISA ± standard errors of the means (SEM) (*n* ≥ 10). Differences in shedding were analyzed using the Mann-Whitney *U* test or by comparing the proportions of total events with >300 CFU using the Fisher exact test. *, *P* < 0.05; ***, *P* < 0.001; ****, *P* < 0.0001; n.s, not significant.

Nasal shedding of pneumococci was quantified in the secretions of pups intranasally (i.n.) colonized at 4 days of life by gently tapping their nares on agar plates. Shedding was measured daily from days of life 5 to 9, the period when the density of shed bacteria is at its peak (18). As previously described (18), a type 23F isolate was shed significantly less than a type 4 isolate, TIGR4 (T4), even though both colonized the nasopharynx at similar levels (Fig. 1A and D). This statistical analysis was based on a comparison of median shedding values as well as the proportion of shedding events that resulted in >300 CFU, a threshold level that correlates with the increased occurrence of intralitter transmission (21).

We postulated that strain-to-strain differences in shedding are attributed to differences in CPS type, genetic background, or both. To assess the contribution of CPS type, we tested an isogenic capsule switch derivative of the T4 isolate expressing a 23F capsule (T4^23F^) or a previously described 23F isolate expressing a type 4 capsule (23F^4^) (23). T4^23F^ was shed less than T4, suggesting that the native CPS of T4 is important for its increased shedding (Fig. 1Bi). When the 23F isolate expressed the type 4 CPS in the construct 23F^4^, the proportion of high shedding events increased compared to that of the 23F isolate (Fig. 1Bii). Importantly, both of the capsule switch constructs colonized at levels similar to that of their parent isolate (Fig. 1D). This suggested a contribution of type 4 CPS to robust shedding and supported the hypothesis that CPS type affects shedding.

Next, we assessed the contribution of genetic background to shedding by comparing strains of different genetic backgrounds expressing the same capsule type (T4 versus 23F^4^ and 23F versus T4^23F^). Median shedding values were similar between T4 and 23F^4^ (Fig. 1Ci). There was a small difference in median shedding between 23F and T4^23F^ (Fig. 1Cii), although there was no difference in proportions of high shedding events for these strains. Together, these findings suggested that CPS type is a more important factor for shedding than genetic background.

We also considered whether the capsule switch strains might express amounts of CPS different from those expressed by their parent isolate and the potential for CPS amount to be a factor in strain-to-strain variation in shedding. A sensitive capture enzyme-linked immunosorbent assay (ELISA) was used to compare total cellular CPS levels among strains of the same CPS type (24). The 23F^4^ derivative expressed significantly less type 4 CPS than the T4 isolate, whereas T4^23F^ and 23F expressed similar amounts of type 23F CPS (Fig. 1E). Thus, in this study, we took into account possible effects of CPS amount as well as type.

### Capsule type affects shedding of strain T4.

**FIG 2:**
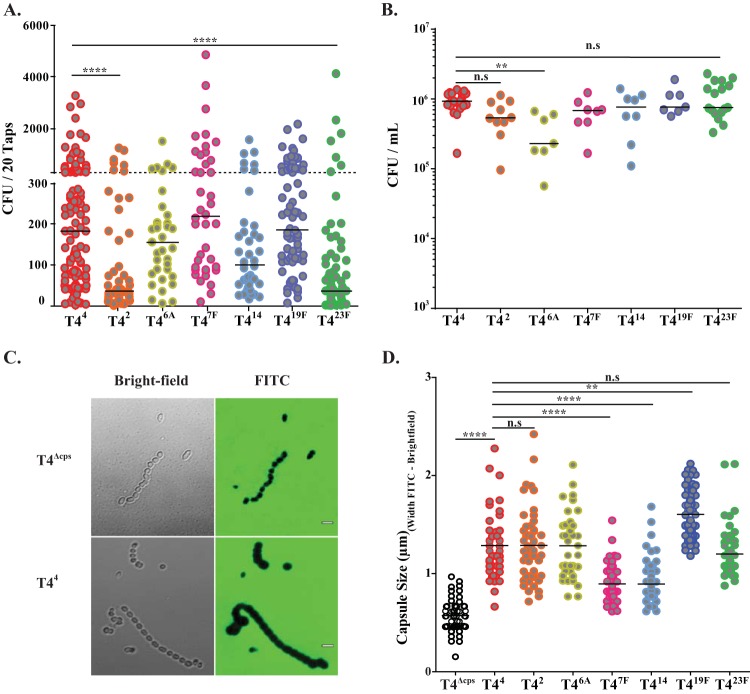
Differences in shedding among isogenic pneumococcal capsule switch constructs. (A) Pups were challenged i.n. at 4 days of age with capsule switch constructs expressing the CPS type shown (indicated in superscript). Bacteria shed daily from nasal secretions were collected and quantified on days 5 to 9. Median values are indicated, and each symbol represents the CFU measured from a single pup. The dashed line represents the 300-CFU threshold described in Results. (B) Colonization density of each construct in cultures of URT lavage fluids obtained from pups at 9 days of age. Median values are shown. (C) Representative bright-field (left) and fluorescent (right) images of T4^Δ*cps*^ and the corrected mutant T4^4^ in the presence of FITC-dextran. Scale bar, 2 µm. (D) Capsule sizes for isogenic capsule switch variants of T4 as determined by the width of bacteria excluded by FITC-dextran. Each symbol represents the calculated relative capsule size for an individual cell, with the median value shown. Each strain was analyzed in three independent experiments. **, *P* < 0.01; ****, *P* < 0.0001; n.s, not significant.

To further examine the role of CPS type on shedding, we tested six additional capsule switch derivatives in an otherwise-isogenic T4 background. Constructs were generated by transforming a capsule-deficient mutant of T4 (T4^Δ*cps*^) with genomic DNA from a clinical isolate of the designated CPS type and then screening for the colony morphology of an encapsulated strain as previously described (25). Capsule switch transformants were then backcrossed into T4^Δ*cps*^ to minimize the possibility of any confounding effect of other genetic loci. A type-4-CPS-expressing corrected mutant of T4^Δ*cps*^ (T4^4^) was also constructed. Pups were inoculated and their shedding was assayed as described above. In addition to T4^23F^, T4^2^ was shed significantly less than T4^4^ (Fig. 2A). The reduced shedding of T4^2^ and T4^23F^ could not be attributed to reduced colonization density (Fig. 2B). Construct T4^6A^ was shed at a level equivalent to T4^4^, even though it colonized at a significantly lower density.

It was not possible to use the quantitative capture ELISA to compare CPS amounts across the capsule switch constructs since the antibodies used were CPS type specific. Instead, a modified fluorescein isothiocyanate (FITC)-dextran exclusion assay was employed to test the possibility that the differences in shedding were attributable to variation in capsule layer thickness (26, 27). T4^2^ and T4^23F^, the constructs that were shed less than T4, expressed capsules similar in size to those of T4^4^ (Fig. 2C and D). We also observed that several constructs either expressed thicker (T4^19F^) or thinner (T4^7F^, T4^14^) capsules than T4^4^, but none of these constructs showed a difference in median shedding or colonization density compared to that of T4^4^. Additionally, there was no difference in bacterial aggregation or average chain length between the different constructs. We concluded that CPS type affects shedding independently of small differences in capsule thickness or colonization density.

### The effect of capsular polysaccharide levels on pneumococcal shedding.

**FIG 3:**
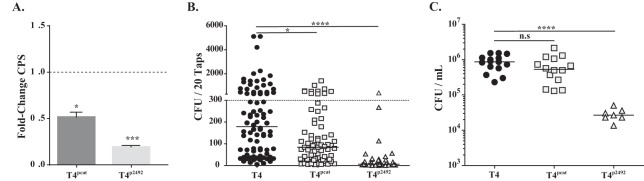
Role of capsule amount in pneumococcal shedding. (A) T4 *cps* promoter switch constructs were compared to their parent (T4) for relative expression of type 4 CPS using a quantitative capture ELISA ± SEM (*n* = 8). (B) Pups were challenged i.n. at 4 days of age with the indicated construct, and bacteria shed daily from nasal secretions were collected and quantified from days 5 to 9. Median values are indicated, and each symbol represents the CFU measured from a single pup. The dashed line represents the 300-CFU threshold described in Results. (C) Colonization density of each promoter switch construct in cultures of URT lavage fluids obtained from pups at 9 days of age. Median values are shown. *, *P* < 0.05; ****, *P* < 0.0001, n.s, not significant.

The *cps* promoter region has considerable sequence variability among pneumococcal isolates and impacts levels of CPS expression (28–30). To examine the contribution of CPS amount in shedding, we tested constructs of the same type that express different levels of CPS. We switched the *cps* promoter region of T4 with either a previously described weaker promoter (to generate T4^*pcat*^) (31) or by replacing the T4 promoter with a *cps* promoter from another isolate shown to express less CPS (to generate T4^p2492^) (28). The amount of CPS produced by these otherwise-isogenic strains was evaluated using a quantitative capture ELISA. Consistently with prior reports of these promoters (28, 31), the T4^*pcat*^ construct expressed ~50% less CPS than T4, whereas T4^p2492^ expressed ~20% of T4 CPS levels (Fig. 3A). We also tested promoter mutants previously identified as expressing increased levels of CPS (28) but were unable to identify any that expressed at least 2-fold-more CPS (data not shown).

We then assessed whether the promoter mutations in T4^*pcat*^ or T4^p2492^ impacted shedding and colonization. Both mutants had reduced shedding compared to T4 (Fig. 3B); however, T4^p2492^ also showed a large defect in colonization compared to the colonization of the parent, T4 (Fig. 3C). As T4^*pcat*^ colonized similarly to the wild type, these data suggest that small changes in the level of CPS expression do not effect pneumococcal colonization but may impact shedding. In contrast, a major reduction in the level of CPS expression results in less shedding, which is likely due to greatly reduced colonization density, as with a *cps*-deficient mutant (18).

### Contribution of capsule type and amount to pneumococcal transmission.

**FIG 4:**
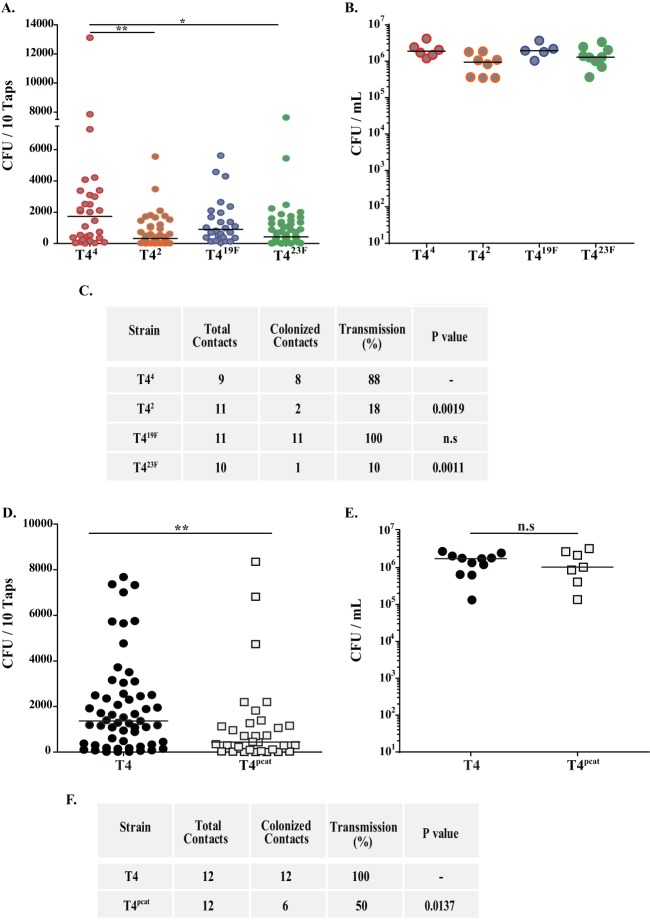
Role of capsule type and amount in pneumococcal transmission. (A) Pneumococcal shedding of isogenic capsule switch constructs in the setting of influenza A virus coinfection. Pups were challenged i.n. at the age of 4 days with the indicated isogenic capsule switch construct. At the age of 9 days, pups were challenged i.n. with influenza A virus and bacteria shed daily in nasal secretions were collected and quantified on days 10 to 14 of age. Median values are indicated, and each symbol represents the CFU measured from a single pup. (B) Colonization density of each isogenic capsule switch construct in cultures of URT lavage fluids obtained from pups at the age of 14 days. Median values are shown. (C) Summary of the transmission rate for isogenic capsule switch constructs from colonized index pups at the age of 4 days to naive contact pups in the same litter. All pups were coinfected with influenza A virus at 9 days of age, and the transmission rate was determined by the number of contact pups colonized by *S. pneumoniae* at the age of 14 days. (D) Pneumococcal shedding of strain T4 and a *cps* promoter mutant (T4^*pcat*^) in the setting of influenza A virus coinfection. Pups were challenged, and shedding was monitored as described above. (E) Colonization densities for T4 and T4^*pcat*^ determined at 14 days of age as described above. (F) Summary of transmission data for strains T4 and T4^*pcat*^ during influenza A virus coinfection. *, *P* < 0.05; **, *P* < 0.01. The proportions of colonized contact pups were compared using the Fisher exact test.

Next, we determined whether decreased shedding in capsule switch derivatives of T4 would lead to a reduction in pup-to-pup transmission. Because rates of intralitter transmission are limiting in pneumococcal mono-infection, we used coinfection with influenza A virus (IAV) as a means of increasing shedding and transmission, as previously described (19, 21, 32). One in three mice per litter was designated an index pup and colonized with pneumococci on day 4 of life; on day 9 of life, all pups in the litter were infected with IAV. Shedding was assessed from days 10 to 14 of life, after which the mice were euthanized and nasal lavages cultured to assess transmission from index to contact pups. The T4 capsule switch mutants that shed less in the mono-infection experiments, T4^2^ and T4^23F^, also showed reduced shedding in the setting of IAV coinfection compared to T4^4^ (Fig. 4A). This contrasted with T4^19F^, which shed robustly during both mono- and coinfection. All constructs colonized at high density (Fig. 4B). Transmission from index to contact pups correlated with shedding: constructs that shed poorly (T4^2^ and T4^23F^) also transmitted at a decreased rate, whereas constructs that shed well (T4^4^ and T4^19F^) showed significantly higher rates of transmission than T4^2^ and T4^23F^ (Fig. 4C). We also examined whether CPS amount affects transmission rates. As only T4^*pcat*^ colonized robustly (Fig. 3C), we compared it to T4 to determine the effect of an ~50% decrease in CPS expression on shedding and transmission in the setting of IAV coinfection, as described above. T4^*pcat*^ showed significantly reduced shedding (Fig. 4D) and transmission (Fig. 4F) compared to those of the parent strain (T4). These results provided further evidence for the correlation between levels of shedding and transmission rate and the impact of both CPS type and amount.

### Effect of capsular polysaccharide type and amount on interaction with mucus.

**FIG 5:**
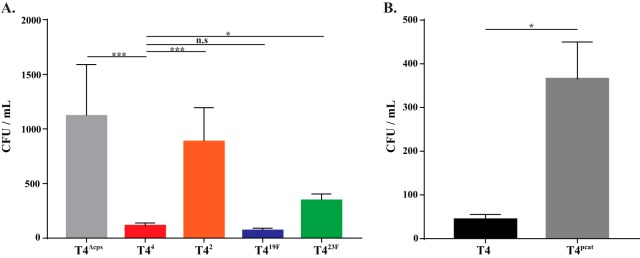
Capsule type and amount affect mucus association. (A) Comparison of levels of binding of a capsule-deficient mutant of T4 (T4^Δ*cps*^) (gray bar) and constructs expressing the CPS type shown (indicated in superscript) to immobilized bovine submaxillary gland mucin. Adherent bacteria were quantified following a 60-min incubation at 30°C. (B) Levels of binding of T4 and T4^*pcat*^ to immobilized bovine submaxillary gland mucin were compared as described above. Values are means of three independent determinations in triplicate ± SEM. *, *P* < 0.05; ***, *P* < 0.001; n.s, not significant.

The mucus layer covering the epithelium of the nasopharynx might provide a barrier for the escape of bacteria to allow their transit to a new host. The expression of CPS has been shown to inhibit pneumococcal interactions with mucus (6). Therefore, we quantified the effect of CPS on pneumococcal escape from mucus using an *in vitro* solid-phase binding assay with URT mucin, the principal component of mucus (6, 33–35). The unencapsulated mutant T4^Δ*cps*^ showed significantly increased binding to bovine submaxillary mucin compared to that of the corrected T4^4^ construct (Fig. 5A), confirming previous results (6). Capsule switch constructs of T4 that showed reduced shedding and transmission, T4^2^ and T4^23F^, also showed increased binding to mucin compared to that of the controls T4^4^ and T4^19F^ (Fig. 5A). Furthermore, T4^*pcat*^, the T4 mutant expressing reduced CPS levels, demonstrated increased mucin binding (Fig. 5B). Thus, strains expressing specific CPS types (such as type 4 or 19F) and larger amounts of CPS are bound less rigorously by mucins and therefore are better able to escape mucus entrapment in the URT. Taken together, these results suggest a correlation between the ability to escape mucin binding and high pneumococcal shedding, which may result in increased transmission of these strains.

## DISCUSSION

A number of functions have been attributed to the capsule of the pneumococcus. The thick layer of CPS shields the organism from recognition by opsonins, such as complement and antibody, and inhibits binding to mucus, which would otherwise lead to URT clearance by mucociliary flow (6, 36, 37). The immunodominance of CPS is thought to have driven its diversification into at least >95 antigenically and structurally distinct types that differ in effectiveness for each of these functions (38). In this report, we used a previously described infant mouse model of nasal colonization, shedding, and transmission (in the context of IAV coinfection) that recapitulates many of the key features of human infection (18, 19, 21). This model was used to test isogenic constructs that varied only in CPS type to show its effect on pneumococcal shedding (without impacting colonization density). The effect of CPS type on shedding correlated with differences in bacterial binding to URT mucins, the abundant polymer-like glycoproteins of mucus. CPS types with decreased shedding showed great binding affinity for mucin, an interaction that might block the organism’s release from mucus and thereby prevent its exit from the host nasopharynx. Moreover, the effect of CPS type on shedding correlated with the frequency of host-to-host transmission. Our results add further evidence for the association between high numbers of shed bacteria and the frequency of transmission events from close contacts. Together, our data demonstrate that pneumococcal types that are more robustly bound by mucin are shed less and are transmitted poorly.

Epidemiology studies have shown that only a subset of over >95 serologically distinct serotypes of *S. pneumoniae* are prevalent in the human population (7, 8, 39). Their advantage may be due to CPS type or differences in genetic backgrounds between strains of the same serogroup. Our observations on the effect of CPS type on transmission frequency may explain, at least in part, why certain pneumococcal types are more or less prevalent in the population. It would be difficult, however, to translate the results for any given type tested in mice to transmission among humans. An example to consider is isolates of type 23F, which are commonly carried in the human population (especially prior to the introduction of PCV) but were shed and transmitted poorly in our study. In this regard, it is well known that CPS blocks opsonization in both mice and humans, but the specific CPS types that are most effective in this process differ by host species (40). Numerous other CPS-dependent effects may be responsible for the epidemiological differences among types in natural transmission; these may include CPS-mediated differences in colonization density, carriage duration, or survival of the pneumococci *ex vivo*.

Because of high rates of genetic exchange, pneumococci are genetically heterogeneous. Our findings suggest that strains may differ in transmissibility and that CPS type is a more important factor than genetic background. We previously identified at least one non-CPS factor, the pore-forming toxin pneumolysin, that induces mucosal inflammation in the URT, which is permissive for high levels of shedding (41). In the current study, we assessed the effect of capsule switch constructs on local inflammation in the nasopharynx and found no significant differences in interleukin 1β (IL-1β) expression or neutrophil influx (data not shown), two key host indicators of acute inflammation, suggesting that type-specific differences in shedding are due to another mechanism. Other unknown pneumococcal products likely contribute to shedding and transmission, but, as is the case for pneumolysin, these may show limited strain-to-strain variability.

We also showed that the amount of CPS affects pneumococcal shedding and transmission. This is not surprising, as strains with thicker capsules are more effective at repelling mucus, and in this study, they also showed less binding to URT mucin, which was associated with increased shedding and transmission. Differences in CPS levels have been shown to result from allelic variation in the *cps* promoter and regulation by phase-variable control of DNA methylation (28, 42). Fluctuations in CPS amount might alter the balance between more or less adhesive phenotypes that impact whether a bacterial cell is retained within or expelled from the mucus layer. Weinberger et al. proposed that a more simple biochemical structure of CPS leads to a large capsule that protects the pneumococcus from phagocytosis by human neutrophils (9). As both of the capsule switch variants T4^2^ and T4^23F^ shed less than T4^4^, we hypothesized that this might be due to their CPS structure. However, we were unable to show a correlation between CPS size and shedding, as both T4^2^ and T4^23F^ had capsules with a thickness similar to that of T4^4^ yet shed less.

As almost all CPS types are negatively charged (due to the presence of uronic acid, phosphate, or pyruvate groups), we reasoned that charge might play a role in escape from negatively charged, heavily sialylated mucins (43, 44). However, neutrally charged CPS variants (types 7F and 14) of T4 shed as well as the T4^4^ construct with negatively charged CPS. It remains unclear, therefore, if there are physical characteristics of the highly heterogeneous CPS structure that directly contribute to the observed interactions with mucin. It is possible that changes in CPS type or amount affect the exposure of underlying pneumococcal adhesins or reveal positively charged bacterial surface features involved in interaction with the mucus layer (45). SP_1492 has been proposed as a surface adhesin that binds with specificity to mucin, including the bovine submaxillary mucin used in this study (46, 47). Relative exposure of SP_1942 or other putative mucin-binding proteins might be dependent on CPS type and amount. A possible requirement for transmission is for shed bacteria to survive outside the host. It has been reported, however, that capsule-deficient strains survive equally well if grown *in vitro* in human saliva and are as desiccation tolerant as an encapsulated strain (48, 49).

Capsule expression is critical for pneumococcal survival within the host. Capsule-deficient strains colonize poorly, and both epidemiological studies and *in vitro* assays support the conclusion that capsule type is more important than genetic background in invasive infection (7, 39, 50, 51). Here we add to our understanding of the contribution of the capsule to the pneumococcus by demonstrating that both CPS type and amount are important for its ability to transmit from host to host.

## MATERIALS AND METHODS

### Ethics statement.

This study was conducted according to the guidelines outlined by National Science Foundation animal welfare requirements and the *Public Health Service Policy on Humane Care and Use of Laboratory Animals* (52). The New York University Medical Center IACUC oversees the welfare, well-being, and proper care and use of all vertebrate animals.

### Growth conditions and strain construction.

Pneumococcal strains were grown statically in tryptic soy (TS) broth (Becton, Dickinson) at 37°C. Upon reaching the desired optical density at 620 nm (OD_620_), cells were washed and diluted in sterile phosphate-buffered saline (PBS) for inoculation. For quantitative culture, serial dilutions were plated on TS broth-streptomycin (200 µg/ml) agar supplemented with either 5% sheep blood or catalase (6,300 U/plate; Worthington Biochemical Corporation) and incubated overnight at 37°C with 5% CO_2_. A streptomycin-resistant derivative of the type 4 strain TIGR4 (T4), P2406, was used throughout the study (18).

The construction of a capsule-deficient mutant of T4 (T4^Δ*cps*^) containing a previously described “sweet Janus” cassette in the *cps* locus and the corrected mutant, T4^4^, was described previously (18, 53). Capsule switch variants of T4 were constructed by transforming T4^Δ*cps*^ with genomic DNA obtained using a MasterPure DNA purification kit (Illumina) from *S. pneumoniae* isolates expressing type 2, 6A, 7F, 14, 19F, or 23F capsule, with selection on TS agar plates supplemented with streptomycin (200 µg/ml) and 10% (wt/vol) sucrose (25). Colonies appearing smooth were picked and confirmed for capsule type by either an immunoblot assay using anti-CPS monoclonal antibody (MAb) (kindly provided by M. Nahm, University of Alabama at Birmingham) or a positive quelling reaction using pneumococcal typing sera (Statens Serum Institut, Denmark). DNA was purified from that isolate and used to retransform (backcross) the unencapsulated T4^Δ*cps*^ strain. The serotypes of back-transformed strains were reconfirmed as described above.

Strain P2492 with a modified promoter in the TIGR4 *cps* locus (T4^p2492^) was constructed in a two-step process. In the first step, we PCR amplified the Janus cassette that replaced the *cps* promoter using genomic DNA from strain TH4702 (28) with primers pr7399 (5′-TTCCTGACGAGAAGGTAGTCAATAA) and pr7344 (5′-GTCTAGATGGACATTCCCTACTGGG) and transformed P2406 (T4) with selection of transformants on TS broth plates supplemented with kanamycin sulfate (500 µg/ml), generating strain P2489. In the second step, P2489 was transformed with a PCR product generated using primers pr7399 and pr7344 on a DNA template from strain TH7054 containing an in-frame type 23F CPS promoter inserted upstream of the capsule locus (28). Transformants were selected on TS broth plates supplemented with streptomycin (200 µg/ml) and confirmed via PCR (28). P2480 (T4^*pcat*^) was constructed by transforming P2406 with genomic DNA obtained from strain 4000 (T4 *pcat cps*) (31) and selecting for transformants on TS broth plates supplemented with chloramphenicol (2.5 µg/ml) (31).

### Shedding and colonization in infant mice.

C57BL/6J mice were obtained from The Jackson Laboratory (Bar Harbor, ME) and bred and maintained in our conventional animal facility. The pups were housed with their dam (mother) for the duration of the experiment and gained weight like uninfected animals.

Four-day-old pups were given an intranasal inoculation without anesthesia containing ~2,000 CFU of *S. pneumoniae* suspended in 3 µl of PBS, as described previously (21). Shedding was quantified by gently tapping the nares (20 taps/pup) on a TS agar plate supplemented with streptomycin (200 µg/ml) to prevent the growth of contaminants and by spreading the secretions over the agar surface with a sterile cotton-tipped swab. To measure colonization density, we euthanized pups at the age indicated in the figures by CO_2_ asphyxiation followed by cardiac puncture. The URT was lavaged with 200 µl of sterile PBS from a needle inserted into the trachea, and fluid was collected from the nares. The limit of detection in lavage fluids was 33 CFU/ml.

To determine the effect of influenza A virus (IAV), we inoculated colonized pups at the age of 9 days i.n. with IAV/HKx31 (2 × 10^4^ 50% tissue culture infective doses [TCID_50_]) in 3 μl and measured shedding from day 10 to day 14 of life as described above.

### Transmission in infant mice.

The pneumococcal transmission model with IAV coinfection was described in previous studies (18, 21). Briefly, one in three pups in the litter was randomly selected and, at day 4 of age, infected with the strain indicated in the figures. These index mice were then returned to the dam and the other uninfected pups (contact mice). At the age of 9 days, all the pups in the litter were inoculated i.n. with IAV/HKx31 as described above. To detect bacterial transmission from the index to contact pups, we euthanized all pups at the age of 14 days and cultured nasal lavages.

### ELISA for quantifying capsular polysaccharide.

Pneumococcal cell lysates were assayed for CPS by capture ELISA as described previously (24). Briefly, strains to be tested were grown to an OD_620_ of 1.0, spun down, and resuspended in 1 ml PBS. Samples were sonicated for 30 s in total (5 s on, 15 s off). Immulon 2HB plates (Thermo Scientific) were coated with type-specific rabbit antiserum (Staten Institut, Denmark) at a dilution of 1:5,000 and incubated overnight at room temperature (RT). The plate was washed 5 times with wash buffer (10 mM Tris, pH 7.4, 0.02% NaN_3_, 150 mM NaCl, 0.05% Brij) and incubated with serial dilutions of sonicated bacteria for 2 h at RT. Samples were detected with an anti-CPS MAb at a 1:300 dilution for 24 h with gentle agitation at 4°C. After the plates were washed 5 times with wash buffer, MAb binding was detected with a goat anti-mouse antibody conjugated to alkaline phosphatase (Sigma-Aldrich; A3688-1 Ml) at a 1:10,000 dilution for 2 h at RT. The plate was washed 5 times before being developed with phosphatase substrate (Thermo Scientific; product number 34068) for 20 min and read at an OD_415_.

### FITC-dextran exclusion assay to measure capsule thickness.

The capsule thicknesses of T4 and its capsule switch derivatives were determined by measuring the zone of exclusion of 2,000-kDa fluorescein isothiocyanate (FITC)-dextran (Sigma; FD2000S-100MG) based on a modification of the method of Gates et al. (26, 27). Strains were grown in 5 ml of TS broth until they reached an OD_620_ of 0.5. After centrifugation at 4,000 × *g* for 5 min, the pellet was resuspended in 5 ml of PBS. To measure the size of encapsulated pneumococci, 100 µl of bacterial suspension was mixed with 20 µl FITC-dextran (10 mg/ml in water). Fluorescent and transmitted light images were collected with a Zeiss LSM 880 laser-scanning confocal microscope with a Plan-Apochromat, 100×-objective, 1.46-numerical-aperture lens. To measure capsule thickness, ImageJ (54) was used to compare the width of the bacteria in the transmitted light image based on intensity profile to the width of the fluorescence excluded by the capsule based on the intensity profile.

### Mucin-binding assay.

Mucin obtained from the bovine submaxillary gland (Calbiochem) was partially purified using PD-10 columns (GE Healthcare) and resuspended in PBS at a concentration of 10 mg/ml as described previously (35). The binding assay was carried out as described elsewhere (6, 34, 35), with the following modifications. Briefly, 96-well MicroWell PolySorp flat-bottom plates (Sigma-Aldrich) were coated with 10 µg/100 µl of semipurified mucin centrifuged at 250 × *g* for 3 min and incubated at 37°C overnight. Afterward, the plate was gently washed 3 times with 100 µl of PBS. Bacterial cultures were grown to mid-log phase (OD_620_, ~0.5), diluted in PBS to a density of 10^5^ CFU/ml, and applied to the plate by centrifugation at 250 × *g* for 3 min at RT. Adherence was determined by incubation at 30°C for 60 min, followed by 15 washes with 100 µl of PBS to remove unbound bacteria. To quantify adherent bacteria, the wells were treated with 200 µl of PBS–0.001% Triton X-100 (Ameresco) for 30 min and mixed vigorously, and 100 µl of sample was plated in triplicate. There was no significant bacterial adherence in the absence of mucin.

### Statistical analysis.

All statistical analyses were performed using GraphPad Prism 7.0 (GraphPad Software, Inc., San Diego, CA). Unless otherwise specified, differences were determined using the Mann-Whitney *U* test (comparing two groups) or the Kruskal-Wallis test with Dunn’s postanalysis (comparing multiple groups).
